# Intrinsically disordered regions in the NLRP family of receptors act as regulatory hubs for inflammasome condensation and activation

**DOI:** 10.3389/fimmu.2026.1793320

**Published:** 2026-09-01

**Authors:** Teresa B. Nava-Ramírez, Cesar L. Cuevas-Velazquez, Alejandra A. Covarrubias, Enrique Rudiño-Piñera, Leonor Pérez-Martínez, Gustavo Pedraza-Alva

**Affiliations:** 1 Departamento de Biología Molecular de Plantas, Instituto de Biotecnología, Universidad Nacional Autónoma de México, Cuernavaca, Morelos, Mexico; 2 Centro de Investigación en Biotecnología, Universidad Autónoma del Estado de Morelos, Cuernavaca, Morelos, Mexico; 3 Laboratorio de Bioquímica Estructural, Departamento de Medicina Molecular y Bioprocesos, Instituto de Biotecnología, Universidad Nacional Autónoma de México, Cuernavaca, Morelos, Mexico; 4 Laboratorio de Neuroinmunobiologia, Departamento de Medicina Molecular y Bioprocesos, Instituto de Biotecnología, Universidad Nacional Autónoma de México, Cuernavaca, Morelos, Mexico

**Keywords:** NLRPs, IDRs, Inflammasome, Inflammation, biocondensates, phase transition

## Abstract

Inflammasomes are multiprotein complexes that orchestrate immune responses to pathogenic and sterile insults by regulating the maturation of inflammatory cytokines and pyroptotic cell death. While inflammasome activation is well-characterized at the biochemical level, the mechanisms governing the spatial and temporal assembly of these complexes remain poorly understood. Here, we uncover a critical role for intrinsically disordered regions (IDRs) in activating NLRP1, NLRP3, and NLRP14 inflammasomes. Through structural prediction analyses, we identify IDRs within these receptors that harbor post-translational modification sites essential for inflammasome assembly and function. Notably, disease-associated mutations in NLRP1 and NLRP3 occur within these IDRs, underscoring their functional relevance in inflammatory disorders. Our computational analysis suggests that IDR-mediated phase separation may drive inflammasome condensation at the perinuclear membrane, serving as a sensor for cellular stress, as stress signals may change their conformation, through post-translational modifications, and thus their interaction capacity. Furthermore, inflammasomes lacking IDRs in their NLRPs may rely on interactions with chaperone or adapter proteins containing IDRs for proper assembly. These insights provide a new framework for understanding the regulation of inflammasomes, suggesting that targeting the dynamics of phase transitions could open novel therapeutic avenues for treating inflammatory and autoimmune diseases.

## Introduction

Inflammation plays a pivotal role as a biological response mechanism restoring homeostasis following various detrimental insults, ranging from pathogenic invasions to physical injuries. This intricate response involves the recognition of pathogens and damage-associated molecular patterns by a class of receptors known as pattern recognition receptors (PRRs), which include toll-like receptors (TLRs) and NOD-like receptors (NLRs), among others. Upon engagement with their respective ligands, these receptors initiate a cascade of events leading to the expression of genes encoding inflammatory cytokines such as TNF, IL-6, IFNγ, IL-18, and IL-1β. While TNF, IL-6, and IFNγ are rapidly secreted upon synthesis, IL-1β and IL-18 require proteolytic cleavage by inflammatory caspases such as caspase-1 and caspase-11 to attain their mature and active cytokine forms. This cleavage event requires a secondary signal, triggered by various factors, including cell membrane damage, endosomal injury, oxidative stress, or cytosolic DNA, whether of foreign (from bacterial or viral sources) or endogenous (genomic or mitochondrial) origin (Reviewed in (1)). The detection of this secondary signal is mediated by a class of receptors known as NLRP receptors (Nucleotide-binding Oligomerization Domain, Leucine-rich Repeat, and Pyrin domain-containing). Upon activation, these receptors undergo a conformational change that promotes their oligomerization and interaction with caspase-1, either directly or via the adapter molecule ASC. This interaction leads to the formation of a multiprotein complex known as the inflammasome. The formation of this complex facilitates the self-activation of caspase-1 to its active form, thereby initiating the production of inflammatory cytokines or triggering a form of inflammatory cell death known as pyroptosis (Reviewed in (1)).

Activation of the inflammasome by various stimuli, including excess energy, β-amyloid peptide, cholesterol, and uric acid, contributes to the perpetuation of chronic inflammation. This sustained inflammatory state can give rise to the development of various pathologies, including type 2 diabetes, neurodegenerative disorders such as Alzheimer’s disease, gout, and even cancer (Reviewed in (2)). Despite our ever-evolving understanding of these processes, the exact mechanisms governing inflammasome assembly and activation remain incompletely understood.

Recent studies have revealed a common factor in the activation of the NLRP3 inflammasome in response to diverse insults: the efflux of potassium ions (K^+^) (3). Additionally, variations in cell volume and exposure to hyper-osmotic signals, such as high salt concentrations, can trigger inflammasome assembly and activation (4). The mechanism by which a decrease in cytosolic K^+^ concentrations or changes in cellular volume cause inflammasome components to relocate to a specific area of the perinuclear membrane, leading to the formation of a macromolecular protein complex that promotes inflammation, remains a mystery.

Biomolecular condensates, dynamic and membrane-less subcellular compartments formed through phase separation or phase transition, have emerged as crucial players in key cellular processes (Reviewed in (5)). These condensates arise from weak yet multivalent interactions between proteins and/or nucleic acids, favoring compartmentalization. This segregation of specific molecules and reactions within the crowded cellular milieu plays a pivotal role in various cellular processes, including gene expression, signal transduction, and stress responses, by enhancing reaction rates and enabling localized functions that promote cell survival (Reviewed in (6)). Consequently, the dysregulation of phase transitions and condensate formation has been linked to various diseases, notably neurodegenerative disorders such as amyotrophic lateral sclerosis and frontotemporal dementia (7).

In recent years, the study of biomolecular phase transitions and the role of proteins containing intrinsically disordered regions (IDRs) have attracted considerable attention (Reviewed in (8)). These IDRs, which lack a stable tertiary structure under physiological conditions, exhibit high conformational flexibility and can interact with various molecular partners. This dynamic nature enables them to contribute to the formation of biomolecular condensates (reviewed in (5)). Proteins that contain IDRs are often found in condensates due to their ability to undergo liquid-liquid phase separation (8). The interactions these proteins engage in can range from weak and transitory to strong and specific, creating a diverse spectrum of molecular compositions within these structures (9).

Given the multi-protein nature of the inflammasome and its specific perinuclear assembly within the cell, we hypothesized that phase-transition processes and IDRs in the NLRP family members, ASC, and inflammatory caspases might play pivotal roles in sensing cellular physicochemical changes triggered by noxious stimuli, thereby leading to inflammasome activation and inflammation. Nonetheless, this intriguing possibility has received limited attention.

Here, we explore the role of IDRs in inflammasome formation and activation through an in-depth analysis of predicted protein structures using AlphaFold. Our findings revealed the presence of IDRs in several members of the NLRP family of receptors. Specifically, we focus our attention and discuss the potential roles of these IDRs in the function of NLRP1, NLRP3, and NLRP14. Our data demonstrate that the IDR within the NLRP1 and NLRP3 inflammasome encompasses sequences containing amino acid residues that undergo post-translational modifications crucial for inflammasome formation and activation. Additionally, we observed mutations in the NLRP1 receptor correlated with Vitiligo-associated multiple autoimmune disease susceptibility 1, and in the NLRP3 receptor correlated with inflammatory diseases, such as familial fever syndrome, occurring within the identified IDRs. In the case of NLRP14, the IDR we uncovered may regulate its interaction with other proteins, facilitating oocyte fertilization. Our findings suggest that the assembly of inflammasomes lacking IDRs in their NLRPs may be governed by interactions between NLRPs and chaperone or adapter proteins containing IDRs.

## Methodology

### Prediction of IDRs in the NLRP protein family

To ascertain the presence of intrinsically disordered regions (IDRs) within the NLRPs protein family, we analyzed the amino acid sequences of human NLRPs family proteins obtained from Uniprot (NLRP1- Q9C000, NLRP2-Q9NX02, NLRP3- Q96P20, NLRP4-Q96MN2, NLRP5-P59047, NLRP6-P59044, NLRP7 Q8WX94, NLRP8-Q86W28, NLRP9- Q288C4, NLRP10-Q86W26, NLRP11- P59045, NLRP12-P59046, NLRP13-Q86W25, NLRP14- Q86W24). An *in silico* analysis was performed employing three disorder prediction tools: the DisEMBL server, IUPRED, and MobiDB (10–12). We employed MobiDB to corroborate the results, as it calculates a disorder consensus score by considering the outcomes of various predictors and their variants (such as Espritz, IUPred, DisEMBL, GlobPlot, etc.) (12). For the DDX3X family disorder prediction, we employed the database D2P2. These predictors assess the disorder status of each amino acid residue within a protein, providing a disorder probability value ranging from 0.0 to 1.0. Regions with a value ≥0.5 are typically classified as IDRs.

### Prediction of protein structures by AlphaFold

Since there are no available crystallographic structures of the NLRPs monomers in the Protein Data Bank, we obtained predicted structures of the NLRPs monomers, including their disordered regions, using the AlphaFold2 platform. These models are used for all the figures except the **Supplementary Figures 2**, **3**, and **10**. Structural models of NLRP1, NLRP3, and NLRP14 in both their phosphorylated and non-phosphorylated states were generated using the AlphaFold 3 server. The predicted structures were subsequently aligned and compared with the corresponding AlphaFold 2 models using PyMOL (The PyMOL Molecular Graphics System, Version 3.0, Schrödinger, LLC). Phosphorylation sites incorporated into the models were retrieved from the GlyGen and PeptideAtlas databases.

### Determination of liquid-liquid phase separation propensity index

To assess the potential for liquid-liquid phase separation and the likelihood of protein condensate formation within the NLRPs, we employed FuzDrop (13). This computational tool calculates the probability of an amino acid sequence to undergo liquid-liquid phase separation (pLLPS). Proteins with pLLPS values greater than or equal to 0.60 are considered capable of spontaneously undergoing liquid-liquid phase separation. Additionally, the program predicts the probability of each amino acid residue participating in interactions that promote condensate formation, enabling the identification of specific regions within the protein sequence with the highest likelihood of undergoing liquid-liquid phase separation (13).

### Sequence and structure alignments of NLRPs with IDRs

To assess the conservation of IDRs within the NLRPs, we conducted two types of alignments: one for amino acid sequences and another for protein structures.

For the amino acid sequence alignment, we utilized the sequences of human NLRP1, NLRP3, and NLRP14 as references. To compile a diverse set of sequences, we conducted a BLAST search on the NCBI BLAST server and obtained 250 different Chordata sequences. These sequences were aligned using the ClustalW algorithm implemented in MEGA software (14). Manual curation was performed to refine the alignment.

The previously obtained monomer 3D structures of human NLRPs were used for the structural alignments against the 3D models generated by AlphaFold2. Additionally, we selected two or three proteins from different animal species for which the monomeric structure was predicted using AlphaFold2. To superpose these 3D structures, we employed the POSA server, a tool designed for multiple alignments of protein coordinates (15).

### Determination of NLRPs interaction zones with other proteins

To identify amino acid sequences of NLRP1, NLRP3, and NLRP14 with a high probability of interaction with other proteins, we utilized the InterProSurf server. This server predicts the likelihood that the surfaces of a protein structure can engage in various types of interactions with other proteins (16). The protein 3D models of NLRP1, NLRP3, and NLRP14 obtained through AlphaFold2 were employed for this analysis. Amino acids with the highest probability of interaction were highlighted in red, while those with a medium likelihood of being involved in protein-protein interactions were marked in green.

To identify potential interaction partners, protein association networks were generated using the STRING server (17). We obtained interaction networks for human NLRP1, NLRP3, and NLRP14. For subsequent molecular docking experiments, we selected the proteins ASC, TxNIP, and TBK1 as their interactions with NLRP1, NLRP3, and NLRP14, respectively, have been experimentally demonstrated.

### Molecular docking

We conducted molecular docking analyses to assess potential interactions between the IDRs of NLRP1, NLRP3, and NLRP14 with ASC, TxNIP, and TBK1, respectively. For this purpose, we utilized the HDock server. We chose this server because it predicts binding complexes between molecules, such as proteins, using a hybrid docking strategy. This strategy enables the seamless integration of experimental data, such as protein–protein interface information and SAXS data, to refine docking poses and enhance the reliability of the results. Only interaction complexes involving the IDRs of NLRP1, NLRP3, and NLRP14, with a docking score value less than -200 and a confidence score value greater than 0.7, were selected. These criteria indicate a high likelihood of interaction, as determined by the HDock algorithm (18). To further evaluate the robustness of the predicted interaction patterns, complementary protein-protein docking analyses were performed using ClusPro (19).

To investigate mutant variants located within the IDRs of NLRP1, NLRP3, and NLRP14 and associated with specific pathologies, we searched the dbSNP database from NCBI. Subsequently, the PremPs server was used to predict how these mutations might affect the structure of NLRP proteins. PremPs evaluates the effects of individual mutations on protein stability by calculating quantitative changes in the Gibbs free energy. The protein structures obtained from AlphaFold2 for NLRP1, NLRP3, and NLRP14, along with the selected mutations within the IDRs, were used to determine changes in the Gibbs free energy, while the changes in the structure were corroborated by analyzing and comparing with the regions of the structures with available experimentally determined 3D structures deposited in the PDB. To confirm the effect of these mutations, we used the available crystallographic structures in the PDB of some NLRPs (PDB entry 7ALV for NLRP3, PDB entry 4N1L for NLRP14, and PDB entry 1PN5 for NLRP1). The mutations resulting in significant changes in NLRP protein structures (considering a highly destabilizing ΔΔGexp ≥ 1.0 kcal mol-1 or highly stabilizing ΔΔGexp ≤ -1.0 kcal mol-1) were analyzed by molecular docking to assess their interaction potential.

## Results and discussion

### NLRPs contain intrinsically disordered regions predicted within their structural composition

We analyzed the NLRP gene family encompassing NLRP 1–14 in humans to ascertain the presence of IDR within their sequences. We identified IDRs in 11 NLRPs (1, 3, 5, 6, 7, 8, 9, 10, 11, 13, 14) (**Table 1**). These regions exhibited disorder scores of ≥ 0.5, as determined by the IUPRED and MobiDB predictors, supporting their computational classification as predicted IDRs; this threshold is standard in the field, but not definitive, and disorder predictions are inherently probabilistic rather than experimentally confirmed. Long IDRs were defined as those containing ≥30 residues, whereas short IDRs contained between 5 and 30 residues (20) (**Supplementary Figure 1**). For further characterization, we focused on NLRP1, NLRP3, and NLRP14 because NLRP1 and NLRP3 play key roles in initiating inflammatory responses to diverse stimuli, whereas NLRP14 has been implicated in regulating the cellular responses to DNA.

**Table 1:** Intrinsically disordered regions in human NLRPs.

Protein	IDR	Sequence	Region aa	Domain
**NLRP1**	IDR1	AGHSPSFPYSPSEP	90-113	After PYD
	IDR2	LPSSPDHESPSQESPNAPTSTAVLGSWGSPPQPSLAPREQEAPGTQWPLDETSGIYYTEIREREREKSEKGRPPWAAVVGTPPQAHTSLQPHHHP	160-254	Polar residues/disorder
	IDR3	VMTPTEGLDTGEMSNSTSSLKRQRLGS	991-1017	After LRR6/disorder
**NLRP2**	IDR1	NKRKPLSLGITRKERPPLDVDEMLERFKTEAQAFTETKGNVICLGKEVFKGKKPDK	101-156	After PYD
**NLRP3**	IDR1	DEPKWGSDNARVSNPTVICQEDSIEEEWMGLLEYLSRISIC	90-130	After PYD
	IDR2	QEREQELLAIGKTKTCE	181-197	FISNA
	IDR3	NMPKEEEEEEKEGRHLDMVQCVLPSSSHAACSHGL	686-720	Acidic Loop
**NLRP5**	IDR1	MKVAGGLELGAAALLSASPRALVTLSTGPTCSILPKNPLFPQNLSSQPCIKMEGD	1-56	N terminal
	IDR2	RDDMKRHSPEDPEATMTDQGPSKEKVPGISQAVQQDSATAAETKEQEISQAMEQEGATAAETEEQEISQAMEQEGATAAETEEQGHGGDT	142-232	After PYD/Disorder
**NLRP6**	IDR1	APEEAMGPAEEPEPGRARRSDTHT	157-181	After PYD/Disorder
	IDR2	PEVTEGAKGLEDTEEPEEEEEGEE	585-614	After LRR1/Disorder
**NLRP7**	IDR1	VQEIDNPELGDAEEDSELAKPGEKEGW	97-123	After PYD/Disorder
**NLRP8**	IDR1	MSDVNPPSDTPIPFSSSSTHSSHIPPW	1-23	N- terminal
	IDR2	PTPHPPDFTGKSDCLSQINP	1029-1048	C- terminal
**NLRP10**	IDR1	GIQMNNVSFKIKHSNEKKSQSQNLFSVKSSLSHGPKEEQKCPSVHGQKEGKDNIAGTQKEASTGKGRGTEETPKNTYI	578-655	C-terminal/Disorder
**NLRP12**	IDR1	RDTPPGGPSSLGNQSTCLLEVSLVTPRKD	96-124	After PYD
**NLRP13**	IDR1	MNFSVITCPNGG	1-12	N-terminal
	IDR2	ENVQTQELQDPTQEDLEMLEAAAGNMQTQGCQDPNQEELDELEEETGNVQAQGCQDPNQEEPEMLEE	107-173	After PYD
**NLRP14**	IDR1	MADSSSS	1-7	Before PYD/Disorder
	IDR2	IGPDDAKAGETQEDQEAVLG	102-121	After PYD/Disorder

Nucleotide-binding oligomerization domain, Leucine rich Repeat and Pyrin domain containing from 1 to 14.

AlphaFold 2 was selected for the structural prediction of the NLRPs owing to the absence of experimentally resolved full-length monomeric structures for these proteins. The comparison between structural models generated by AlphaFold 2 and AlphaFold 3 revealed that AlphaFold 2 more accurately preserved the extended and flexible characteristics expected in IDRs. In contrast, AlphaFold 3 tended to generate more compact conformations in these segments. Accordingly, when comparing the structures predicted by both versions of AlphaFold, we observed that the IDR regions were more flexible in the AlphaFold 2 models than in the AlphaFold 3 models (**Supplementary Figure 2**).

Although AlphaFold 3 represents a significant advance in biomolecular structure prediction, several recent studies suggest that AlphaFold 2 may retain specific advantages for analyzing IDRs (21, 22). AlphaFold 2 has been shown to more consistently capture disorder propensity through low-confidence predictions and extended flexible conformations, thereby avoiding the overinterpretation of disordered segments as stable folded structures. Recent comparative analyses indicate that AlphaFold 3, despite improvements in complex prediction and the reduction of certain structural uncertainties, may introduce artificially compact conformations or structural artifacts within highly dynamic IDRs, potentially complicating the interpretation of intrinsically disordered states. Furthermore, AlphaFold 2-derived confidence metrics, such as pLDDT scores and predicted solvent accessibility, have been widely employed as proxies for disorder prediction and conditional folding behavior (21, 22).

Given that our study focuses on the identification and comparative analysis of flexible and disordered regions rather than on determining stable atomic-resolution conformations, and that AlphaFold 2 provides a robust and well-characterized framework that remains broadly supported in the current literature for evaluating IDRs the AlphaFold 2 models were selected for subsequent analyses.

**Figure 1:**
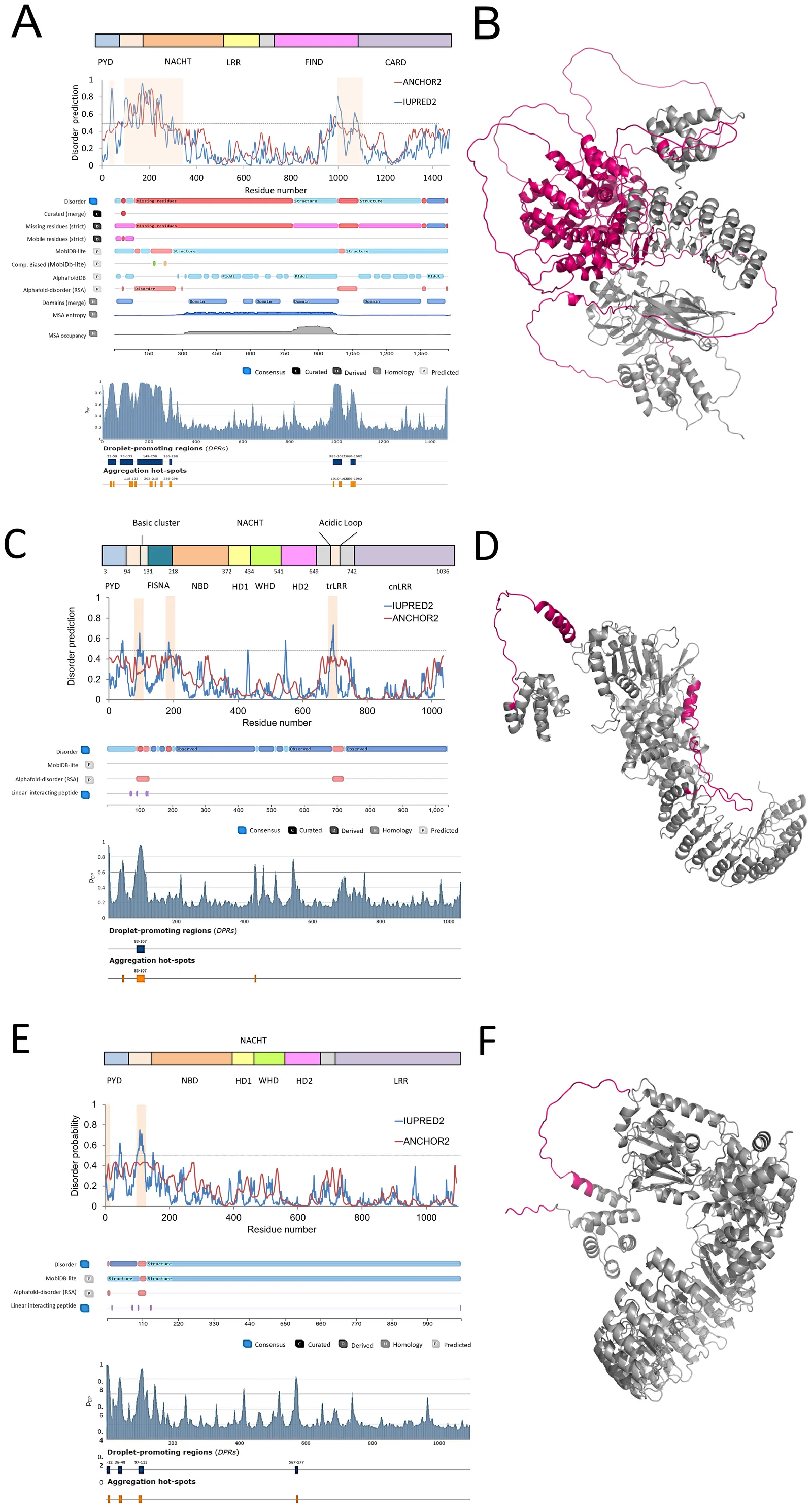
NLRP1, NLRP3, and NLRP14 contain intrinsically disordered regions. **(A)** From top to bottom, diagram of structure, disorder score predicted by IUPRED2 (IDRs are highlighted with translucent orange blocks), and MobiDB, and diagram of LLPS scores predicted by FuzDrop for NLRP1. **(B)** Human NLRP1 structure predicted by AlphaFold2. In all structures, the predicted IDRs by MobiDB are colored in magenta. **(C)** Diagram of the linear structure of human NLRP3, disorder score predicted by IUPRED2 and MobiDB, and diagram of LLPS scores predicted by FuzDrop. **(D)** Human NLRP3 structure as predicted by AlphaFold. **(E)** Diagram of the linear NLRP14 structure, disorder score predicted by IUPRED2 and MobiDB, and diagram of the LLPS scores predicted by FuzDrop. **(F)** Human NLRP14 linear structure as predicted by AlphaFold.

In NLRP1, three distinct IDRs were identified: IDR1 spanning amino acid residues 90-113, IDR2 covering residues 160-254, and IDR3 found within residues 991-1017. Interestingly, the identified IDRs were localized near or within functionally defined motifs. Both IDR1 and IDR2 in NLRP1 were localized downstream of the pyrin domain (PYD), while IDR3 was located within the eucine-rich repeats (LRR), specifically within the acidic loop (**Figure 1A**). Accordingly, the available crystallographic structures for NLRP1 (PDB entry 6x6c) exhibit poorly defined regions that lack electron density, including amino acid residues 94–789 and 990-1078 (**Figure 1B**). This supports the predictions from MobiDB and AlphaFold2, which indicate that these regions are structurally disordered and can be considered as IDRs.

For NLRP3, three distinct IDRs were identified: IDR1 spanning residues 90-130, IDR2 covering residues 181-197, and IDR3 within residues 686-720 (**Figure 1C**). As with NLRP1, IDR1 and IDR2 in NLRP3 were positioned downstream of the PYD domain, whereas IDR3 was located within the LRR region, specifically within the acidic loop (**Figures 1C, D**).

Likewise, in NLRP14, we observed two prominent IDRs: IDR1, a 7-amino-acid region located immediately before the PYD domain, and IDR2, spanning residues 95-121 (**Figures 1E, F**), which exhibited remarkable similarity to IDR1 in NLRP3 (**Figure 1F**).

Notably, IDR1 and IDR2 in NLRP1, NLRP3, and NLRP14 exhibited particularly high scores for liquid-liquid phase separation (LLPS), suggesting that these regions may function as hotspots for aggregation (**Figures 1A, C, E**, lower panel). This is significant as it increases the likelihood of interactions with other molecular partners via these disordered regions. Additionally, AlphaFold2 structural predictions suggest that IDR2 in NLRP3 may adopt an alpha-helical conformation, which could facilitate interactions with other proteins (**Figure 1C**).

### The intrinsically disordered regions in NLRP1, NLRP3, and NLRP14 are conserved across chordates

**Figure 2:**
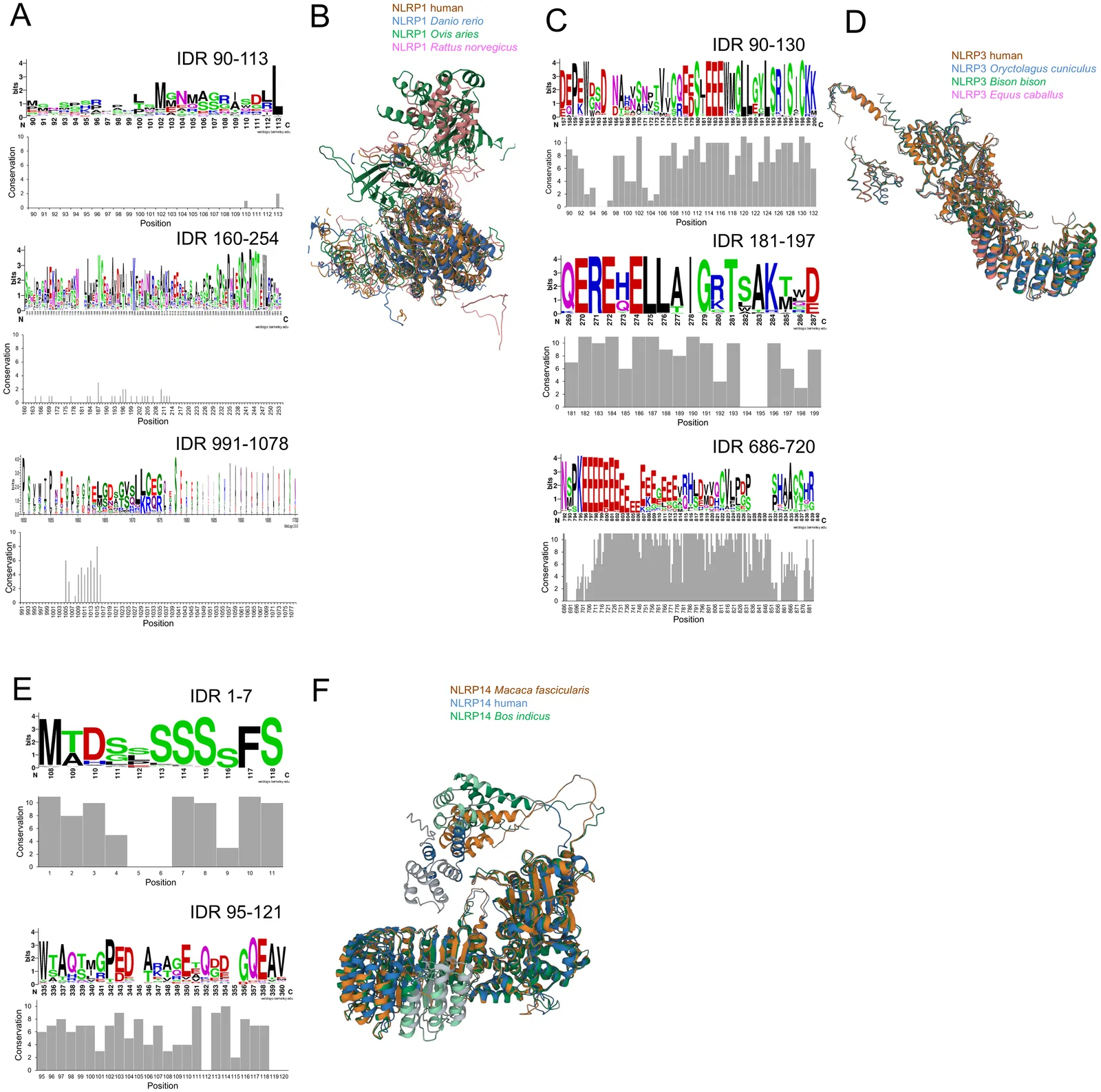
Conservation of intrinsically disordered regions (IDRs) in NLRP1, NLRP3, and NLRP14 across species. **(A)** Logo representation of the consensus sequence of IDRs in NLRP1, derived from the alignment of 250 NLRP1 sequences from chordate species. The IDRs in NLRP1 correspond to IDR1 (90-113), IDR2 (160-254), and IDR3 (991-1017). **(B)** Structural alignment of NLRP1 from human (brown), *Danio rerio* (blue), *Ovis aries* (green), and *Rattus norvegicus* (pink). **(C)** Logo representation of the consensus sequence of IDRs in NLRP3, derived from the alignment of 250 NLRP3 sequences from chordate species. The IDRs in the human sequence correspond to IDR1 (90-130), IDR2 (181-191), and IDR3 (686-720). **(E)** Logo representation of the consensus sequence of IDRs in NLRP14, obtained from the alignment of 250 NLRP14 sequences from chordate species. The IDRs in the human sequence correspond to IDR1 (1-7) and IDR2 (95-121). **(F)** Structural alignment of NLRP14 from human (blue), *Macaca fascicularis* (brown), and *Bos indicus* (green).

To assess the potential significance of these IDRs in NLRP1, NLRP3, and NLRP14 for their activity, we examined the evolutionary conservation of their amino acid sequences and predicted three-dimensional structures. Orthologous sequences corresponding to a 250-amino-acid region encompassing the IDRs were retrieved from chordates using the NCBI database. Sequence conservation analysis revealed that the IDRs of all three proteins are enriched in charged and hydrophilic amino acids, a characteristic feature of IDRs (**Figures 2A, C, E**). The IDR sequences of NLRP3 and NLRP14 exhibited high conservation scores, suggesting that these regions are subject to evolutionary constraints that preserve not only their overall physicochemical properties but also some specific amino acid identities (**Figures 2C, E**). In contrast, the IDRs of NLRP1 displayed lower sequence conservation (**Figure 2A**). Such variability is consistent with the evolutionary behavior of IDRs, which often tolerate extensive sequence divergence because their functions depend on their structural flexibility and overall physicochemical properties, allowing the selection of specific conformations from their ensembles rather than conserved primary sequence (23). To evaluate structural conservation, we analyzed representative NRLP orthologues from several animal species using AlphaFold2 for structure prediction. The predicted structures were subsequently aligned with the POSA server, which revealed a high degree of structural similarity with C-alpha RMSD values ranging from 2.08 to 2.58 Å for NLRP1, NLRP3, and NLRP14 (**Figures 2B, D, F**).

**Figure 3:**
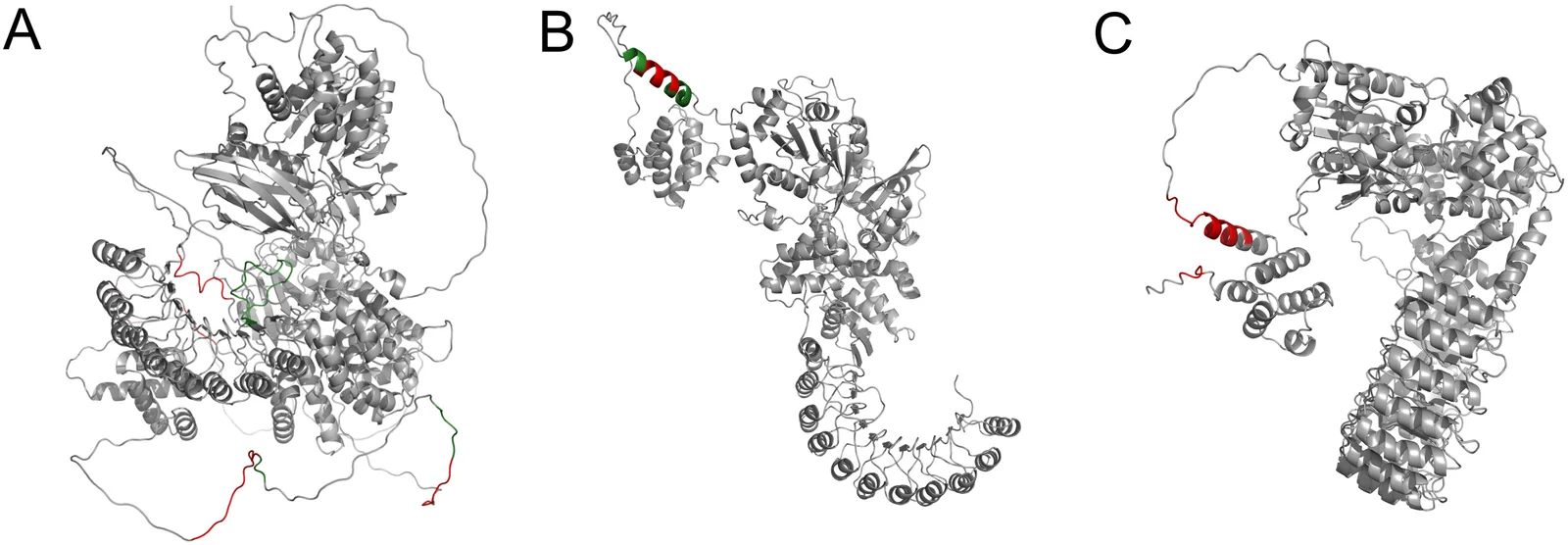
IDRs downstream of the PYD domain of NLRP1, NLRP3, and NLRP14 are potentially involved in protein-protein interactions. The protein-protein interactions prediction was performed using the InterProtSurf server. In these figures, the regions with a high probability of interaction are indicated in red, while those with medium probability are highlighted in green. Predicted structures for human **(A)** NLRP1, **(B)** NLRP3, and **(C)** NLRP14.

Given the remarkable conservation observed in the structure-predicted models and among the amino acid sequences in most of these IDRs, along with their high probability of serving as interaction sites, we assessed the likelihood of these IDRs to engage with other proteins using InterProSurf. Our analysis revealed a significant probability of interaction, particularly in the IDR following the PYD domain of NLRP1, NLRP3, and NLRP14. Thus, these IDRs may play a role in NLRP activity by serving as interaction hubs for various protein targets (**Figures 3A–C**).

### IDRs located downstream of the PYD domain act as central hubs for protein interactions that regulate NLRP activation

**Figure 4:**
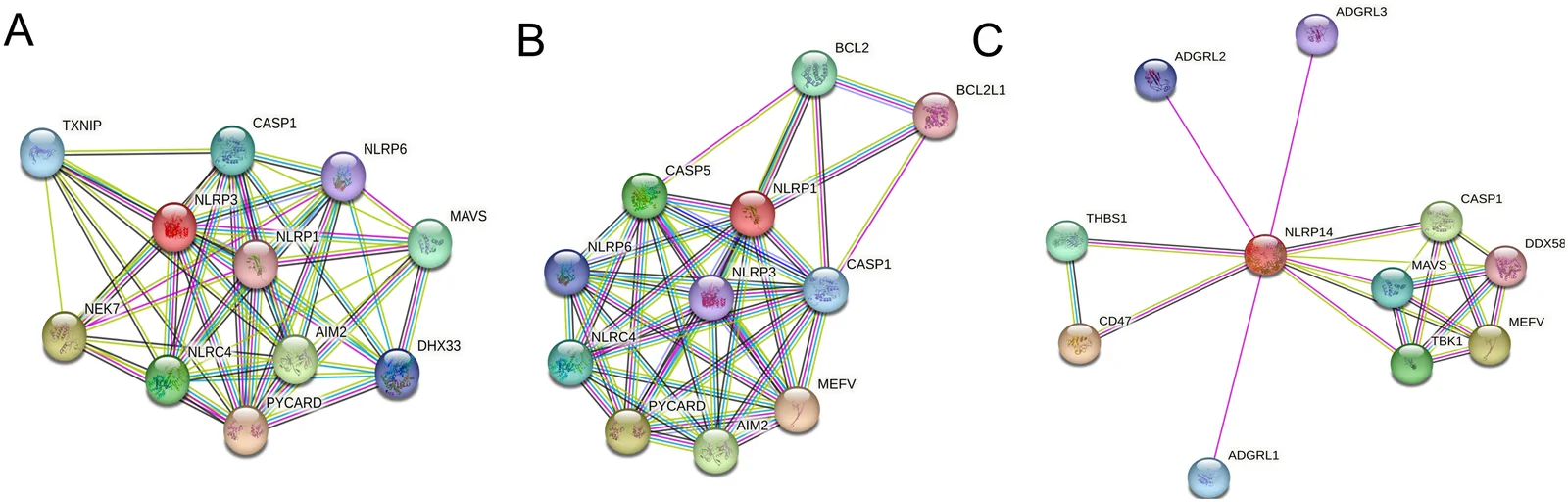
Interaction maps for NLRP1, NRLP3, and NRLP14. The protein association networks were obtained through the STRING database. **(A)** NLRP1 interaction map. **(B)** NLRP3 interaction map. **(C)** NLRP14 interaction map.

To reinforce the possibility of NLRP1, NLRP3, and NLRP14 as nodes of protein-protein interactions, we used the STRING database to generate interaction maps for human NLRP1, NLRP3, and NLRP14 (**Figures 4A–C**). From these maps, we selected BCL2, a key anti-apoptotic protein, recently described to control pyroptosis (inflammatory cell death) by negatively regulating NLRP1 inflammasome assembly and activation (24). Additionally, we chose TXNIP, as this protein has been proposed to interact directly with NLRP3, promoting inflammasome assembly and activation under oxidative stress conditions (25), as well as TBK1, a recently identified NLRP14 interactor involved in oocyte fertilization (26). Next, we evaluated the likelihood of interaction between the IDRs on NLRPs and the selected proteins using molecular docking via the HDock server. The predicted NLRP interaction patterns were further validated using the ClusPro docking server (**Supplementary Figure 3**).

#### NLRP1

NLRP1 is among the best-characterized NLRPs. Microbial ligands capable of activating NLRP1 include muramyl dipeptide (MDP), a component of peptidoglycans produced by Gram-positive and Gram-negative bacteria (27). The murine NLRP1b isoform was reported to be crucial for anthrax-lethal toxin-mediated macrophage cell killing (28), and for regulating IL-18 production in the adipose tissue of obese mice (29). The human ortholog is involved in triggering inflammation in response to skin damage caused by ultraviolet radiation (30). Additionally, hereditary polymorphisms in NLRP1 have been associated with vitiligo and related autoimmune diseases (31).

BCL2 and BCL-X_L_ negatively regulate NLRP1 inflammasome activation by binding to NLRP1 (32). This inhibitory protein-protein interaction requires contact between the BCL2 and BCL-XL loop domains with the LRR domain on NLRP1, as BCL-2 or BCLXL mutants lacking the loop domain are unable to block NLRP1 inflammasome and caspase 1 activation. Similarly, an NLRP1 mutant lacking the LRR is insensitive to the negative regulation imposed by BCL2 and BCL-X_L_ (24, 32). Computational docking analysis predicts that the amino acid residues 700 to 770, located in the NLRP1 LRR, interact with BCL2 in 3 of the 10 best models. Notably, this amino acid sequence was not defined in the crystallographic structure, suggesting high structural flexibility (PDB entry 2YV6). In addition to this region, our computational analysis predicts that the IDR 2 of NLRP1 (amino acid residues 160-254) may also contact BCL-2 (**Supplementary Figure 4**). However, the experimental evidence does not support this possibility; an NLRP1 mutant lacking the first 350 amino acids (which includes the full PYD domain and IDR2) can interact with BCL-2 (**Supplementary Figure 4**). Similar results were observed when we evaluated whether the IDRs in NLRP1 contact BCL-XL (**Supplementary Figure 5**). These data suggest that the LRR in NLRP1 contains a region with remarkable structural flexibility, which could facilitate protein-protein interactions. Although docking is a useful tool for predicting potential molecular interactions, it is important to consider that this methodology can generate false positives due to inherent limitations in prediction algorithms and the representation of protein conformational dynamics (33, 34). Nevertheless, experimental evidence supports the possibility that the LRR region of NLRP1 participates in the interaction with BCL-2 (32), validating our predictions.

**Figure 5:**
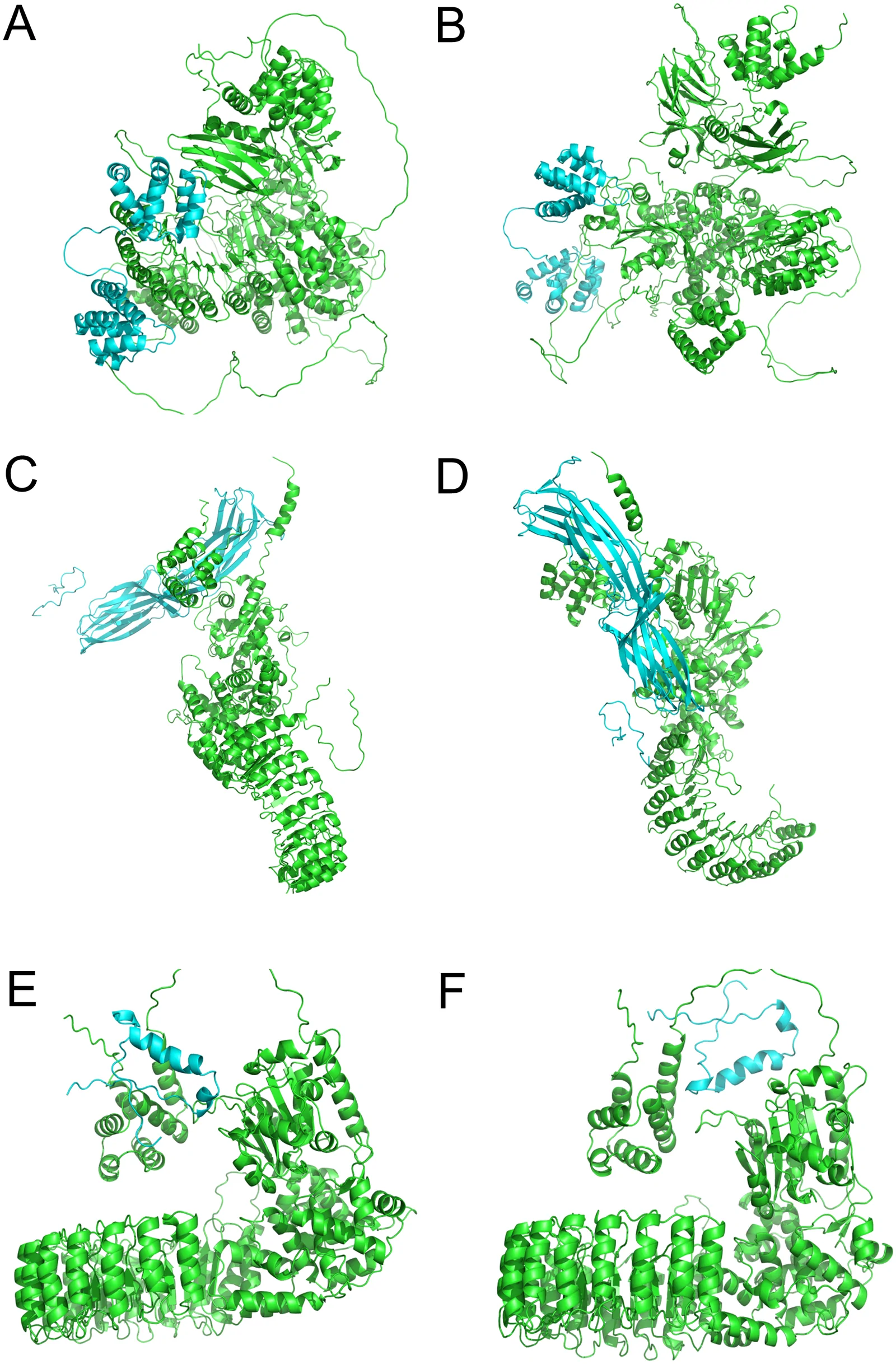
IDRs downstream of the PYD domain of human NLRP1, NLRP3, and NLRP14 could interact with ASC, TxNIP, and TBK1 proteins, respectively. Interaction models were generated via molecular docking using the HDock Server. Models showing a high probability of interaction involving the IDRs were selected for further analysis. **(A)** Model illustrating the interaction between NLRP1 and ASC. Docking score: -259.22, RMSD with ligand: 71.69 Å **(B)** Model demonstrating the interaction between NLRP1 and ASC. Docking score: -255.46, RMSD with ligand: 59.61 Å **(C)** Model illustrating the interaction between NLRP3 and TxNIP. Docking score: -216.4, RMSD with ligand: 89.88 Å **(D)** Model demonstrating the interaction between NLRP3 and TxNIP. Docking score: -215.87, RMSD with ligand: 83.92 Å **(E)** Model depicting the interaction between NLRP14 and TBK1. Docking score: -203.95, RMSD with ligand: 122.84 Å **(F)** Model illustrating the interaction between NLRP14 and TBK1. Docking score: -202.1, RMSD with ligand: 108.91 Å. NRLPs are shown in green, while ligands are colored in cyan.

Given that the adapter molecule ASC (also known as PYCARD) plays a role in NLRP1 activation (35), we also evaluated whether the NLRP1-ASC interactions (**Figure 5A**) involve the IDRs found in NLRP1. In this case, eight models from the top ten predictions showed interactions with the IDR1, IDR2, and IDR3 regions of NLRP1, with RMSD values ranging from 56.37 Å to 79.18 Å, respectively. This RMSD value is very high (>5 Å) and suggests that the predicted conformation differs significantly from the native structure, which could indicate an unreliable prediction. Despite this, we analyzed two models with a high probability of interaction. The first model predicts ASC interacting with IDR1 (**Figure 5A**), while the second predicts ASC interacting with IDR2 (**Figure 5B**). Interestingly, recent data indicate that NLRP1 activation involves the proteolytic processing of the autoinhibitory N-terminal fragment (NT) and the oligomerization of the liberated C-terminal fragment NLRP1^UPA-CARD^ (CT) to drive inflammasome activation. The potential interactions between ASC and the IDR1 of NLRP1 suggest that the released inhibitory N-terminal fragment, which includes the PYD domain and IDR1 during NLRP1 activation, could interact with ASC. This interaction might serve as a mechanism to disengage ASC from the assembled inflammasome, potentially inhibiting caspase-1 activation and IL-1β production, thereby attenuating the inflammatory response. Although this hypothesis requires experimental validation, the idea that a mechanism for reducing the inflammatory response to maintain homeostasis involves the expression of genes encoding proteins solely containing the pyrin domain, a family of human-specific proteins referred to as pyrin-only proteins or POPs (Reviewed in (36)), supports this concept. POP1 inhibits ASC nucleation, impairing inflammasome assembly, caspase-1 activation, IL-1β and IL-18 release, and pyroptosis (37). The amino acid residues in POP1 that interact with ASC have been identified (E13, K21, R41, R48, and D51) (38). Interestingly, all of them map to IDR1 (amino acid residues 1-99) in POP1, distinct from the other four IDRs identified (IDR2: 161-183, IDR3: 553-400, IDR4: 724-799, and IDR5: 913-943, **Supplementary Figure 6**). This supports a direct role for this IDR in NLRP3 inflammasome assembly and the inflammatory response mediated by POP1. Furthermore, the amino acid residues in POP1 that mediate the interaction with ASC are conserved in the PYD domain of ASC, and these residues also facilitate the ASC/NLRP3 interaction (**Supplementary Figure 6A**). This interaction involves the ASC PYD domain and the NLRP3 PYD domain. The fact that POP1 does not interact with NLRP3 suggests that other amino acid sequences are involved in determining specificity among PYD-PYD interactions.

However, whether the other IDRs in POP1 participate in the negative control of other inflammasomes by interacting with their PYD domains and blocking ASC recruitment remains to be determined.

#### NLRP3

The key role of the NLRP3 inflammasome in regulating both acute and chronic inflammation has been well documented. Although K^+^ exit is a common activating signal (3), the precise mechanisms by which diverse stimuli, such as oxidative stress, ATP, nigericin, silica, amyloid β, and mitochondrial DNA, induce NLRP3 inflammasome assembly and activation are poorly understood. Questions such as how K^+^ efflux from the cell promotes NLRP3 inflammasome assembly, which protein sequences (domains) facilitate NLRP3 oligomerization, and which protein interactors are necessary for full activation remain unanswered. Recent data provide some information. Initially, it was believed that the leucine-rich domain, like that in Toll-like receptors (TLRs), played a role in sensing bacterial products and activating NLRP3. However, recent data indicate that the LRR domain is dispensable for NLRP3 inflammasome activation in response to several stimuli (39). Unlike the NLRP1 PYD and the linker domain, the NLRP3 PYD and the linker domain, which contain the IDR1 (residues 90 to 130), are sufficient for NLRP3 activation in response to bacterial stimuli (*Francisella novicida*) and to sterile stimulation (oxidative stress, H_2_O_2_). Meanwhile, the IDR2 (residues 181 to 199) may participate in NLRP3 activation in response to *Listeria monocytogenes*, pore-forming toxins (nigericin), and uric acid crystals. Finally, IDR3 (residues 686 to 720) might assist the NLRP3 inflammasome activation in response to sterile insults, such as H_2_O_2_, nigericin, and uric acid crystals (39). In the context of obesity-induced inflammation, oxidative stress resulting from high glucose levels releases TXNIP from thioredoxin, leading to NLRP3 activation and obesity-induced glucose intolerance (25). However, the mechanism by which TXNIP engages NLRP3 inflammasome activation remains largely unknown. Two models from the top 10 predictions showed TXNIP interactions with the IDR1 and IDR2 of NLRP3, with RMSD values of 89.88 Å and 83.92 Å, respectively (**Figures 5C, D**). These results suggest that, upon oxidative stress, TXNIP may interact with NLRP3 IDR1 and IDR2 to trigger inflammasome assembly.

As mentioned above, K^+^ efflux is a key step for NLRP3 inflammasome activation induced by distinct stimuli (3). Despite extensive investigation, the exact molecular mechanism controlling NLRP3 activation after K^+^ efflux remains unknown. Recent evidence suggests that the mitotic kinase NEK7 licenses NLRP3 inflammasome assembly and activation, acting downstream of K^+^ efflux (40). NLRP3-activating stimuli (ATP, pore-forming toxins, and particulate matter) promoted the NLRP3–NEK7 interaction in a process dependent on K^+^ efflux. Interestingly, NLRP3 interacts with the catalytic domain of NEK7, although NEK7 catalytic activity is dispensable for NLRP3 inflammasome activation (40, 41). Thus, NEK7 is considered a common downstream intermediate for various agonists evoking NLRP3 activation in a K^+^ exit-dependent manner. Overexpression experiments suggest that NEK7 binds to three major surfaces on NLRP3, namely the LRR, HD2, and nucleotide-binding domains (40–42). Our computational analysis predicts NEK7 interactions with the IDR1 (residues 90 to 130) and the IDR3 (residues 686 to 720) of NLRP3 (**Supplementary Figures 7A, B**). Although the LRR and the HD2 may contribute to NEK7 and NLRP3 interactions, point mutants in the LRR reduce but do not impair nigericin-induced IL-1β maturation (42). In contrast, data show that an NLRP3 deletion mutant lacking the LRR domain but retaining amino acid residues 686 to 720 (corresponding to IDR3) remains fully responsive to nigericin, promoting IL-1β production at levels comparable to wild-type NLRP3 (43). These data support the role of the NLRP3 IDR3 in mediating NLRP3-NEK7 interactions and inflammasome assembly downstream of K^+^ efflux. Furthermore, the only evidence suggesting a role for IDR1 in mediating NLRP3/NEK7 interaction is that the NLRP3 mutant lacking the PYD domain, which interacts with NEK7 (40), also lacks four amino acids (90-DEPK-94) from the IDR1 (residues 90 to 130). Nonetheless, NEK7 interaction with IDR3 could favor ASC binding to NLRP3 via IDR1, thereby promoting inflammasome assembly and activation. Supporting this, our structural models also predict that ASC contacts NLRP3 through IDR1, located immediately downstream of the PYD domain; in fact, three of the top ten predicted models showed ASC interacting with NLRP3 via IDR1 (**Supplementary Figures 7A, B**).

In addition to NEK7-NLRP3 interactions, recent data suggest that protein-protein interactions tightly regulate NLRP3 inflammasome assembly and activation. NLRP11 was identified as an essential component of the NLRP3 inflammasome in human macrophages. The lack of NLRP11 impaired NLRP3 inflammasome activation by preventing inflammasome assembly (NLRP3 and ASC oligomerization), thus blunting caspase-1 activation and cytokine release induced by nigericin, silica crystals, and K^+^ efflux (44), without affecting other inflammasomes. The PYD domain of NLRP11 plays a key role in NLRP3 inflammasome assembly and activation. While NACHT-NACHT interactions are known to mediate contacts between NLRP11 and NLRP3, it has been suggested that additional regions are required to provide specificity, given that the NLRP11 NACHT domain also interacts with other NACHT-containing proteins. Our computational analysis predicts that NLRP3 interacts with NLRP11 not only through NACHT-NACHT contacts, but also via multiple amino acid residues in the IDR1 of NLRP3, with the IDR2 of NLRP11, and through interactions between residues in NLRP3 IDR1 and the PYD domain of NLRP11 (**Supplementary Figure 8**).

The location of NLRP3 within the cell is key for inflammasome activation. Proteins such as MASV and mitofusin-2 are involved in the NLRP3 mitochondrial localization in response to RNA viruses (45, 46). Lipids like cardiolipin function as scaffolds mediating NLRP3 mitochondrial localization and activation during mitochondrial stress. Interestingly, a region within the LRR domain (residues 546 to 719) was found to be required for NLRP3 binding to cardiolipin and for mitochondrial localization (47). Due to their low sequence complexity and enrichment in charged residues, IDRs can promote multivalent electrostatic interactions at the surfaces of lipid bilayers (48). Thus, it is possible that IDR3 found in the NLRP3 LRR domain (residues 686 to 720, **Table 1**) mediates cardiolipin binding and NLRP3 mitochondrial localization. Recently, it was reported that NLRP3 interacts with SCAP-SREBP2 complex via its NATCH domain, forming a ternary complex that translocates to the Golgi apparatus adjacent to mitochondrial clusters to facilitate optimal inflammasome assembly (49). Consequently, NLRP3 localization to mitochondria is at least a two-step process involving different NLRP3 domains. The NATCHT domain, through interaction with SCAP-SREBP2, brings NLRP3 to the mitochondrial proximity, and cardiolipin binding via the IDR3, located in the LRR domain, stabilizes NLRP3 mitochondrial localization and inflammasome activation.

Together, our analysis highlights that the NLRP3 IDRs could be key hubs that i) coordinate NLRP3 protein-protein interactions in the resting state, impairing unwanted activation, and ii) promote structural changes allowing activating interactions upon stimulation.

Nonetheless, how K^+^ impairs NEK7 and NLRP11 interactions with NLRP3 in the resting state, thus avoiding unwanted activation, remains to be determined.

#### NLRP14

When considering NLRP function, the onset of inflammation in response to sterile and non-sterile stimuli comes to mind. However, not all NLRPs contribute to inflammation. Recent findings indicate that NLRP14 plays a key role in fertilization.

All cells have specialized proteins that detect cytosolic nucleic acids to defend against infections by initiating innate and adaptive immune responses. The innate response is characterized by the production of type I interferons (IFN-α/β), which help combat invading pathogens (Reviewed in (50)). These inflammatory programs are tightly regulated to prevent unwanted activation against self-nucleic acids.

For example, the cytosolic receptor RIG-I (retinoic acid-inducible gene 1) detects viral RNA by recognizing a unique 5’-triphosphate structure, allowing it to distinguish self from non-self RNA (50). In contrast, cytosolic DNA sensing, mediated by cGAS (cyclic GMP-AMP synthase) and STING (stimulator of interferon genes), does not differentiate between self and non-self-DNA (51).

Activation of these cytosolic sensors and endosomal TLR3 by double-stranded RNA (dsRNA) leads to the engagement of TBK1 (TANK-binding kinase 1). TBK1 then phosphorylates and activates interferon regulatory factors IRF3 and IRF7, which drive the expression of antiviral interferon-stimulated genes (ISGs).

During fertilization, when the oocyte cytosol is exposed to sperm genetic material, the sensing of cytosolic genetic material should be finely regulated to ensure progeny and species propagation. Recently, it was observed that through its interaction with TBK1, NLRP14 impairs the sensing of cytosolic genetic material. NLRP14 interacts with TBK1, targeting TBK1 for ubiquitination and degradation (26). Although the exact domain in NLRP14 that mediates NLRP14/TBK1 interactions was not defined, the PYD and the NATCH domains of NLRP14 interact with the TBK1 kinase domain. The in silico analysis in this work predicts that the IDR found in NLRP14 (residues 102 to 121) may mediate the interaction with TBK1. Two models from the top 10 predictions suggest interactions with the IDR1, with RMSD values of 122.84 Å and 108.91 Å, respectively (**Figures 5E, F**). Consistent with this the NLRP14 NATCH domain alone (residues 176 to 345) was not sufficient to suppress IRF7-mediated promoter activation. Similarly, an NLRP14 mutant lacking the Pyrin domain and some amino acids from the linker region (residues 1 to 100) shows a partial loss of its ability to impair TBK1-mediated IRF7 activation (26). Together, these data indicate that the IDR in NLRP14 mediates the interaction with TBK, allowing fertilization, a hypothesis that remains to be experimentally validated.

In addition to its interaction with TBK1, NLRP14 may attenuate the genetic material sensing response by associating with other members of the signaling pathway, such as IRF3, STING, RIG-I, and MAVS (mitochondrial antiviral signaling protein). Consistent with the fact that none of these proteins are kinases, in silico analysis predicts that their interaction with NLRP14 is mediated not by the IDR but rather through the LRR domain (**Figures 5E, F**).

Interestingly, another member of the NLRP family of receptors has been implicated in regulating the antiviral response. Cui et al. found that NLRP4 (which shares 38% of sequence identity with NLRP14) negatively regulates RNA sensing by targeting TBK1 for ubiquitination and proteasomal degradation (52). Our analysis revealed no IDRs within the NLRP4 protein sequence. Accordingly, previous findings indicate that NLRP4 interacts with the TBK1 kinase domain through its NOD domain (52). This supports the notion that the TBK kinase domain serves as a hub for NLRP interactions. However, the specificity determinants underlying this specificity remain to be elucidated.

In contrast to NRLP14 and NLRP4, the NLRP6 inflammasome, in addition to its role in combating bacterial infection in the gut mucosa, promotes the antiviral response independently of caspase-1 activation (53). It was found that NLRP6 induces the production of type I and III interferons in response to double-stranded RNA. This function of NLRP6 limits intestinal viral infection, specifically against RNA viruses (53). The molecular mechanism by which NLRP6 coordinates the anti-viral response involves recognition of double-stranded RNA through its NATCH domain and interaction with the pre-mRNA-splicing factor and adenosine triphosphate (ATP)–dependent RNA helicase Dhx15, which modulates antiviral immune responses via MAVS. NLRP6 interacts with Dhx15 via its NATCH and NACHT-associated domain (NAD), thus promoting Dhx15 interaction with MVS and the onset of the antiviral response (53). Interestingly, the IDRs we identified in NLRP6 were located in domains involved in both RNA and Dhx15 interaction. NLRP6 IDR1 (residues 157 to 181) and IDR2 (residues 585 to 614) were contained in the NATCH construct used to map NLRP6 interactions with RNA (aa residues 144-400), while only IDR2 (residues 585-614) was contained in the NATCH-NAD construct used to determine the NLRP6 sequences mediating the interaction with Dhx15. These data suggest a role for the NLRP6 IDRs in facilitating NLRP6 protein-protein interactions and double-stranded RNA recognition, a key process for an efficient antiviral response.

Together, these data suggest a role for NLRP IDRs in the cellular response to cytosolic genetic material, inhibiting or promoting the interferon response.

### The IDRs located downstream of the PYD domain of NLRP1, NLRP3, and NLRP14 may act as hubs that integrate stress signals, leading to inflammasome activation and inflammatory response

#### NLRP1

NLRP1 must undergo structural changes to form a functional inflammasome, involving proteasomal degradation of the autoinhibitory N-terminal fragment (NT) and the oligomerization of the liberated C-terminal fragment NLRP1^UPA-CARD^ (CT) to drive inflammasome activation (54). Post-translational modifications of the NLRP1 linker region might trigger these structural changes.

Phosphorylation of NLRP1 at S179 and T180 by the long-splice isoform of MAP3K20 (ZAKα kinase) in response to UVB-driven RNA damage mediates NLRP1 inflammasome activation in human keratinocytes (55). Interestingly, these amino acids are located in the IDR2, along with several residues (S163, S168, S170, S173, and T178) phosphorylated by the MAPKs p38α and p38β downstream of ZAKα, which are necessary for full NLRP1 activation upon UVB-induced damage (55). Moreover, the IDR1 contains two p38α and p38β phosphorylation sites (S93 and S99). These data strongly suggest an essential role for IDR1 and IDR2 in NLRP1 Inflammasome assembly and activation.

Of note, the NLRP1 amino acid sequence between IDR1 and IDR2 is a structured region forming a short alpha helix. This region contains an additional ZAKα motif and p38α phosphorylation sites, suggesting crosstalk between these regions to support NLRP1 activation. This idea is supported by recent results indicating that NLRP1 activation in human bronchial epithelial cells infected with human rhinovirus (HRV) requires its cleavage by the HRV cysteine protease 3Cpro. This protease targets amino acids Q130 and G131, located between the two IDRs (IDR1 and IDR2). This cleavage allows recognition of the autoinhibitory N-terminal fragment (residues 131 to 1212) by the N-terminal glycine degron involving the Cullin ZER1/ZYG11B complex and subsequent clearance by the proteasome (56). The released NLRP1 C-terminal fragment (residues 1213 to 1474) leads to inflammasome activation, caspase-1 activation, inflammatory cell death, and the secretion of inflammatory cytokines, such as IL-18, from infected airway epithelial cells.

Remarkably, an early event during HRV infection of bronchial epithelial cells and bronchoalveolar macrophages is the activation of p38 MAPK (57, 58). Thus, phosphorylation of the amino acid residues in the IDR1 or the IDR2 by p38α may promote conformational changes exposing the 3Cpro cleavage site and allowing NLRP1 processing and inflammasome activation.

This finding aligns with another study, which indicates that NLRP1 phosphorylation at S107 by p38α mediates inflammasome activation in keratinocytes in response to ribotoxic stress and alphaviruses, including Chikungunya virus, Barmah Forest virus, and Ross River virus (59). Phosphorylation at S107 results in NLRP1 ubiquitination by the cullin complex, degradation of its N-terminal region, and recruitment of ASC by the NLRP1 C-terminal region.

Although there are potential MAPK phosphorylation sites in NLRP1 IDR3, no reports indicate that this IDR might be sensitive to phosphorylation by stress-activated kinases. In conclusion, our data suggest that IDR1 and IDR2 might translate cellular stress signals delivered by p38α activation, acting as a unifying signaling hub, into the structural changes that control NLRP1 inflammasome activation to maintain skin and airway mucosal homeostasis.

#### NLRP3

NLRP3 is a crucial component of the innate immune system, and its activation leads to the formation of the NLRP3 inflammasome, a multi-protein complex that plays a vital role in the inflammatory response. The activation of NLRP3 is a multi-faceted process regulated by various signals, including PAMPs, DAMPs, and specific post-translational modifications. The intricate balance of these signals and modifications determines the inflammasome’s activation status, ultimately influencing the inflammatory response.

However, it remains unclear whether NLRP3-specific post-translational modifications alter its structure to inhibit or promote activation, and whether IDRs are involved in these processes.

NLRP3 activation is regulated both positively and negatively by post-translational modifications (PTMs). Phosphorylation at S5 in the pyrin domain inhibits oligomerization of NLRP3 and activation of the NLRP3 inflammasome (60, 61). Similarly, phosphorylation of S295 in mouse NLRP3 impairs its activation (62). In agreement with these results, phosphatase 2A depletion impairs NLRP3 activation, indicating that phosphate removal from these sites is key for licensing NLRP3 inflammasome assembly (60). Dephosphorylation of S5 might promote conformational changes, allowing subsequent PTMs, such as S198 phosphorylation. JNK1-mediated S198 phosphorylation increases inflammasome activation, promoting NLRP3 homo-oligomerization (63). S198 is located at the C-terminal of IDR2 (amino acid residues 181 to 199) in the human NLRP3 protein. Notably, IDR1 (amino acid residues 90 to 137) encompasses the linker region (amino acid residues 91 to 132), whereas IDR2 maps within the FISNA domain (amino acid residues 137 to 208), two distinct structural regions of NLRP3 that are key to allowing the conformational changes required for NLRP3 oligomerization, ASC recruitment, and productive inflammasome assembly (64). The polybasic region of the human NLRP3 protein (136-YRKKYRKYVRSRY-148) at the beginning of the FISNA domain mediates human NLRP3 binding to PtdIns4P in the dispersed trans-Golgi network after K^+^ efflux, promoting the relocalization of NLRP3 and assembly of the NLRP3 inflammasome (64). Interestingly, the phosphorylation of Y136, Y140, Y143, and Y168 by Bruton’s tyrosine kinase (BTK) promotes NLRP3 relocalization, oligomerization, ASC polymerization, and full inflammasome assembly by neutralizing the charge of the polybasic sequence in the FISNA domain, directing NLRP3 Golgi association and inflammasome nucleation (65). Together, these results suggest that phosphorylation of S198 promotes structural changes in the IDR2, allowing deubiquitylation of K496, which in turn exposes the positively charged amino acids in the FISNA domain, rendering them susceptible to BTK phosphorylation and promoting NLRP3 transition to the open active conformation upon K^+^ exit (64). Stimuli that promote NLRP3 inflammasome assembly and activation independently of a drop in intracellular K^+^ concentration, like imiquimod, require IDR1 for NLRP3 activation; however, whether IDR1 in response to imiquimod favors the PTMs required for NLRP3 activation after K^+^ efflux remains to be determined.

It has been reported that phosphorylation at S295 in the NATCH domain by PKA inhibits NLRP3 activation, whereas phosphorylation by PKD promotes activation (66). This implies that S295 phosphorylation requires a specific set of additional PTMs to mediate opposing effects on NLRP3 activation, likely priming the different NLRP3 IDRs to undergo structural changes that dictate NLRP3 inhibition or activation. However, this remains to be elucidated.

NLRP3 ubiquitination also has contrasting effects. While the ubiquitination of K496 maintains NLRP3 in an inactive state, the ubiquitination of K384 and K689 is associated with NLRP3 inactivation. In contrast, the ubiquitination of K567 is necessary for NLRP3 activation. Like the phosphorylation of S295, the ubiquitination of K689 appears to have contrasting roles, as it may also be required for NLRP3 activation (Reviewed by (67)). Interestingly, this residue is in the IDR3. Again, the effect of K689 ubiquitination will be determined by a specific set of PTMs in other NLRP3 IDRs, which will dictate whether the protein adopts an open active conformation or remains in a closed inactive state.

Sumoylation at K204 is also required for NLRP3 activation (67). This residue lies within the FISNA domain, which, as mentioned above, mediates NLRP3 nucleation and activation at the Golgi network. It remains unclear whether sumoylation at this residue enhances the phosphorylation of the Y residues within the FISNA domain to promote NLRP3 interaction with PtdIns4P, or if the phosphorylation of these residues facilitates sumoylation. Nonetheless, these PTMs act through IDRs to drive NLRP3 conformational transitions, enabling a fine-tuned regulation of its activation.

### Phosphorylation at the IDRs may induce conformational changes, potentially modulating their capacity to mediate NLRPs’ intermolecular interactions

To assess whether phosphorylation alters the structure of the predicted IDR, a comparative analysis was performed between the predicted structures of the non-phosphorylated and phosphorylated forms of NLRP1 and NLRP3 at the residues reported to modulate their assembly and function. To this end, the phosphorylated structures of the NLRPs were predicted using the AlphaFold 3 server. The resulting structures were then aligned and compared with the corresponding non-phosphorylated models using PyMOL 3.1. Surprisingly, phosphorylation at those sites did not promote any structural change (Data not shown). Nonetheless, multiple phosphorylation sites have been identified in NLRP1, NLRP3, and NLRP14 and are catalogued in the PhosphoSitePlus server (68), a curated database of experimentally detected post-translational modifications. As mentioned above, NLRP3 contains numerous validated phosphorylation sites distributed across the PYD, NACHT, and LRR domains, including residues such as Ser5, Ser198, Ser295, Ser387, and Tyr861, which have been associated with regulation of inflammasome priming, oligomerization, protein stability, and inflammatory activation (69–71). In contrast, although the functional characterization of phosphorylation events in NLRP1 and NLRP14 remains more limited, several phosphosites have also been reported for these proteins in phosphoproteomic studies and curated datasets, suggesting that post-translational phosphorylation may broadly contribute to the regulation of NLRP family activity, intermolecular interactions, and signaling dynamics (72, 73). The presence of phosphorylation sites within or adjacent to IDRs is particularly relevant, as these flexible regions frequently act as regulatory hubs that facilitate condition-dependent conformational changes and transient protein–protein interactions (74).

Several of the identified phosphorylation sites are localized within the IDRs of the NLRPs (**Supplementary Table 1**). These phosphorylated residues were evaluated in the comparative analysis between the predicted structures of the non-phosphorylated and phosphorylated forms; we observed no apparent structural alterations in NLRP1 upon phosphorylation (**Supplementary Figure 9A**). Accordingly, a computational docking analysis did not reveal significant changes in the predicted interaction patterns relative to the non-phosphorylated structure, with a strong predicted interaction consistently observed at IDR2, located immediately downstream of the PYD domain. In contrast, phosphorylation-associated structural changes in NLRP3 and NLRP14 were observed (**Supplementary Figures 9B, C**). For NLRP3, phosphorylation at Thr659 promoted the formation of an α-helix within IDR3 (**Supplementary Figure 9B**; Table S). In the phosphorylated NLRP3 structure, the newly identified phosphorylation sites within IDR2 (Y136, Y140, Y143, and Y168) did not produce apparent structural changes (**Supplementary Figure 9B**). However, computational docking analysis predicted altered interaction patterns compared to the non-phosphorylated structure. The strongest predicted interactions suggested IDR3, with three of the ten highest-ranked docking models engaging this region. This suggests the structural change in the predicted IDR3 resulting from T659 phosphorylation allows downstream NLRP3 protein-protein interactions (**Supplementary Figure 10**). Similarly, for NLRP14, phosphorylation at Thr101 induced structural changes within IDR1, generating an α-helix (**Supplementary Figure 9C**). Notably, these phosphorylation-associated conformational alterations in NLRP14 reduced the potential interactions involving this IDR as predicted by the HDOCK Server, suggesting that phosphorylation may modulate the interaction capacity of these flexible regions.

### Exploring the impact of mutations in IDRs of NLRPs on protein stability and interaction

**Figure 6:**
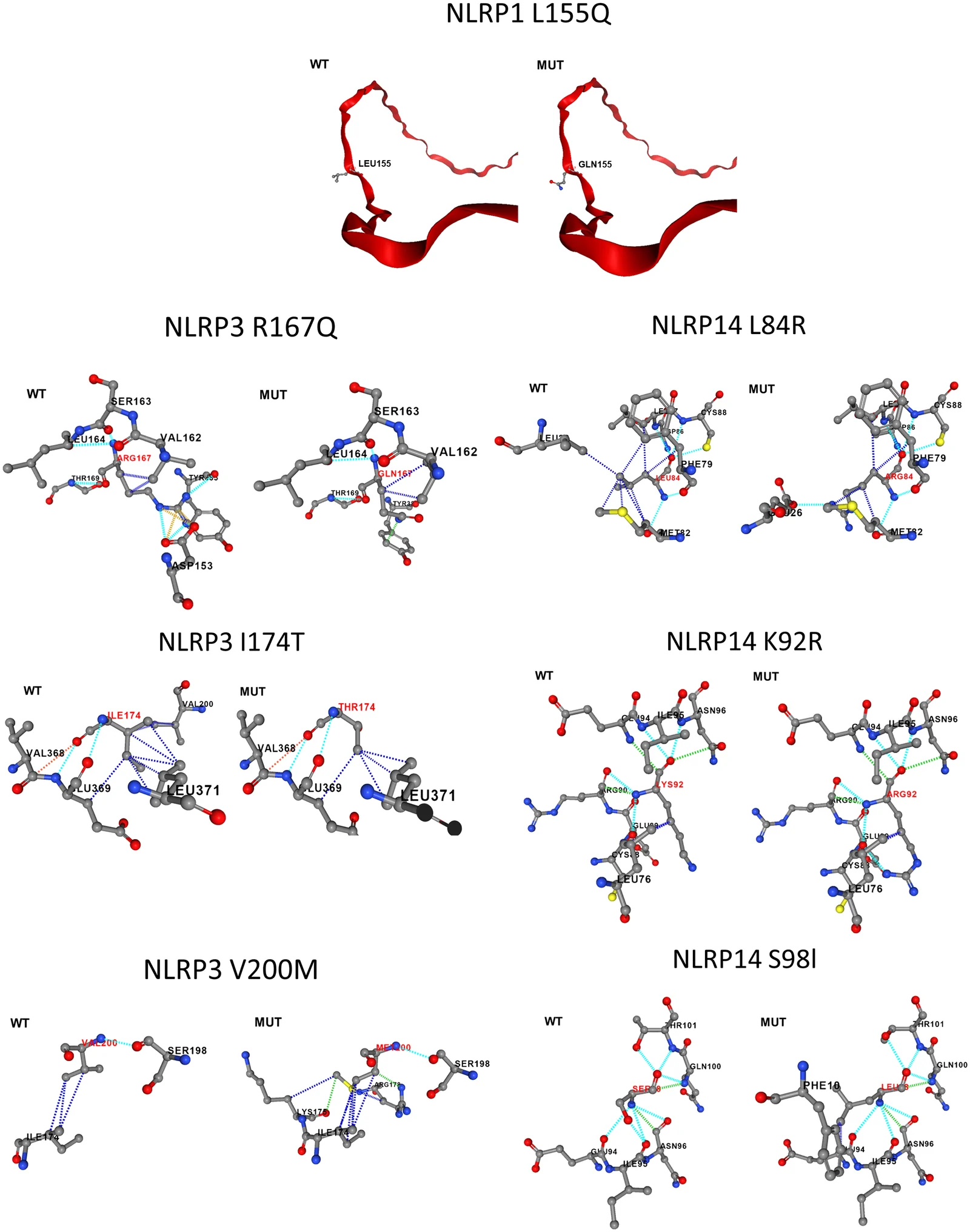
Intramolecular interactions in WT and mutant NLRPs. Relevant segments of wild-type and mutant NLRP proteins are shown in stick representation. The wild-type structure (*left panel*) is compared with the mutant structure (*right panel*). The top panel shows the predicted NLRP1 loop conformation of the wild-type and the L155Q mutant protein, with disordered regions represented as a red ribbon, indicating the location of the mutation. The remaining panels show the schematic representations of the non-covalent interactions between amino acid residues within the IDRs of wild-type and mutant NLRP1, NLRP3, and NLRP14. Interactions are represented by dotted lines and color-coded as follows: hydrogen bonds (purple), ionic interactions (orange), hydrophobic interactions (navy blue), polar interactions (cyan), aromatic interactions (pink), van der Waals interactions (green), and carbonyl interactions (red).

To explore the potential link between mutations within the IDRs of NLRP1, NLRP3, and NLRP14 and human inflammatory conditions, a search in the NCBI database for mutant variants in the IDRs following the PYD domain was conducted, and their associations with specific pathologies were examined (**Table 2**). Upon analyzing the structural alterations induced by these mutations, we found that only the R167Q and I174T mutants of NLRP3 reduced structural stability due to bond disruption with other amino acids (**Table 2**, **Figure 6**). In the case of NLRP3 R167Q, a loss of hydrophobic bonds is observed between Q167 and V162, as well as the loss of ionic and polar interactions with N153 and Y383. In NLRP3 I174T, there is a loss of hydrophobic bonds between T174 and amino acids L371 and V200. On the other hand, in NLRP14, the K92R and S98L mutants enhanced structural stability, whereas the R84L mutant impaired structural stability. However, the fall in ΔΔG values within the range typically considered neutral (≤ 1.0 kcal mol-1 or ≥ -1.0 kcal mol-1) indicates that these mutations are unlikely to induce significant structural changes (**Table 2**, **Figure 6**). Of note, not all mutations affected the NLRP14 ability to interact with ASC, TXNIP, and TBK1.

**Table 2:** Disease-associated mutations within intrinsically disordered regions of NLRP1, NLRP3, and NLRP14.

Proteins	Mutation	IDR	ΔΔG*	Location in structure	Pathology
NLRP1	L155Q	IDR2	0.24	Surface	Vitiligo-associated multiple autoimmune disease 1 (VAMAS1)
NLRP3	C108S	IDR1	0.16	Surface	No pathology reported
	R167Q	IDR2	1.17	Inter	Cryopyrin-Associated Periodic Syndrome
	I174T	IDR2	1.21	Inter	Chronic infantile neurological cutaneous and articular syndrome (CINCA)
	V200M	IDR2	0.0	Surface	Familial cold autoinflammatory syndrome 1 (FCAS1) and Muckle-Wells syndrome (MWS)
	Q705K	IDR3	0.06	Surface	FCAS1
NLRP14	L84R	IDR2	0.25	Inter	No pathology reported. There is greater thermal stability of the pyrin domain.
	D86V	IDR2	0.04	Surface	Spermatogenic failure
	K92R	IDR2	-0.23	Surface	Benign
	S98L	IDR2	-0.37	Surface	No pathology reported

* We consider variants without structural changes whose ΔΔG values are in the range between -0.5 and 0.5 kcal/mol.

In the case of NLRP1, interestingly, three of the gain-of-function mutants A54T, A66V, M77T, associated with skin inflammation and cancer development (75), and the A59P mutant, associated with corneal and mucosal dyskeratosis, are located within the PYD domain, upstream of IDR1 and IDR2. While the molecular mechanism by which these mutants enhance NLRP1 inflammasome activation remains unknown, a role for IDR1 and IDR2 cannot be discarded. To validate the structural changes predicted for the various mutations, the crystallographic structures of the NLRP domains were used as a reference. This analysis revealed similar structural changes, as outlined in **Supplementary Table 2**.

### The DDX3X protein interacts with the IDRs of NLRP3

**Figure 7:**
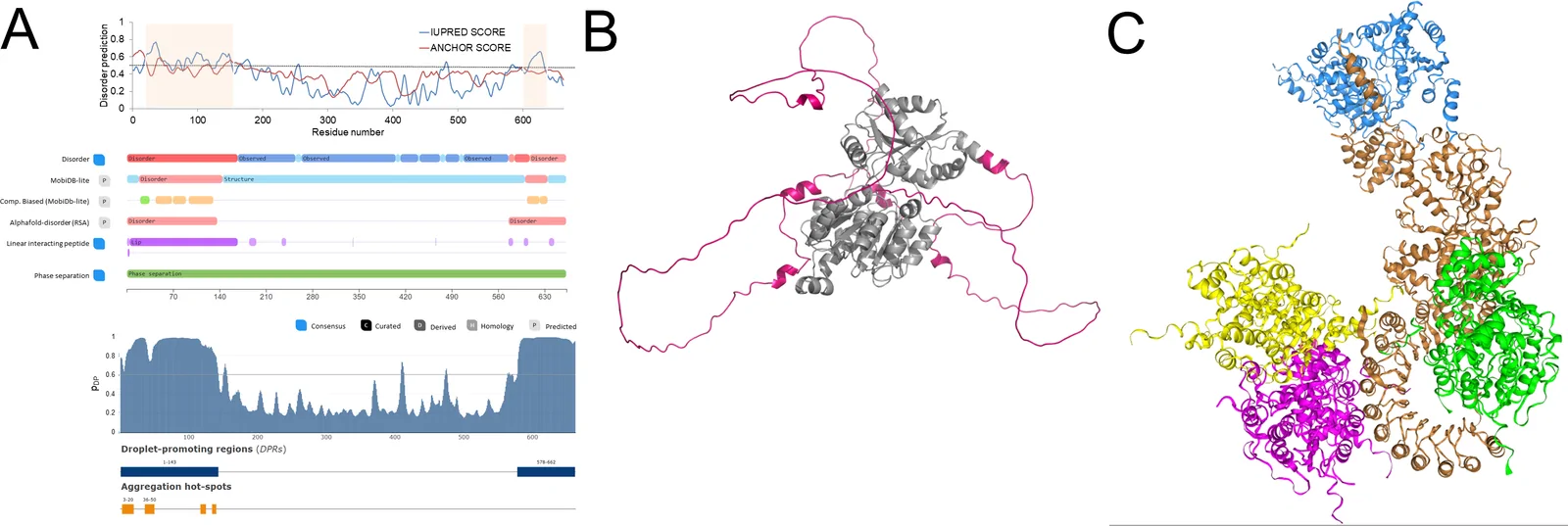
DDX3X IDRs interact with NLRP3 IDRs. **(A)** From top to bottom: schematic diagram of the protein domain structure; disorder scores predicted by IUPRED2 and MobiDB (IDRs highlighted with translucent orange blocks); and LLPS propensity scores predicted by FuzDrop for DDX3X. **(B)** Predicted structure of DDX3X generated by AlphaFold 2. IDRs as predicted by MobiDB are highlighted in magenta. **(C)** Computational docking models of the predicted interaction between NLRP3 and DDX3X, as generated by HDOCK. NLRP3 is shown in brown; each color represents a distinct docking model for DDX3X. The blue DDX3X model predicts interaction with IDR2, located immediately downstream of the PYD domain of NLRP3; the yellow, magenta, and green DDX3X models predict interactions with IDR3, localized within the acidic loop of the LRR region of NLRP3.

Recently, it has been reported that proteins involved in stress granule formation interact with NLRP3, regulating its activation (76); however, the interaction site between the two has not been described. DDX3X is a protein that has an IDR at the N-terminus ranging from residues 1 to 167, and another IDR at the C-terminus ranging from residues 576 to 662 (**Figures 7A, B**). Molecular docking analysis predicted that DDX3X interacts with NLRP3, with four of the top ten predicted models showing a high probability of interaction. DDX3X interacts with NLRP3 primarily through its N-terminal region, which binds to the IDR3 of NLRP3, while the IDR2 of NLRP3 interacts with the C-terminal IDR of DDX3X (**Figure 7C**).

We also evaluated whether the human DDX3X family contains IDRs at their N- and C-terminal regions. Our analysis revealed that all 18 members of the DDX3X family contain IDRs with variable length in their structure (**Supplementary Figure 11**). However, there is no conservation at the amino acid sequence level among these IDRs (data not shown).

## Concluding remarks

Our computational analyses explore the predicted functional significance of IDRs in the NLRP family members, specifically NLRP1, NLRP3, and NLRP14. These IDRs, located downstream of the PYD domain, are structurally conserved across species despite limited sequence similarity. They exhibit high predicted propensity for protein-protein interactions and present features associated with liquid-liquid phase separation. Structural modeling and molecular docking analyses are consistent with a model in which these regions may act as interaction hubs, potentially mediating contacts with key regulatory proteins such as BCL-2, TXNIP, TBK1, NEK7, and ASC. However, all proposed interactions remain computationally predicted and have not been experimentally validated; the distinction between predicted interaction propensity and demonstrated molecular function must be explicitly acknowledged. Through these putative interactions, the IDRs may contribute to inflammasome assembly and the regulation of downstream signaling pathways, although causality remains to be established. It is also acknowledged that not all NLRP family members present IDRs of comparable extent, and that structured NLRPs may rely on alternative regulatory mechanisms, including allosteric regulation within folded domains, chaperone-mediated conformational control (e.g., HSP90, HSP70), and adaptor protein scaffolding, to achieve equivalent functional flexibility; a balanced discussion of these possibilities is warranted.

Post-translational modifications within these IDRs, such as phosphorylation and ubiquitination, may further modulate NLRP activation in response to diverse stimuli, including oxidative stress, K^+^ efflux, viral infection, and UV-induced damage. Furthermore, several mutations found within these regions are associated with altered protein stability and human inflammatory diseases, underlining their physiological relevance.

Together, our computational analyses are consistent with a model in which IDRs in NLRPs may function not merely as flexible linkers but as dynamic regulatory elements that integrate environmental cues and coordinate the structural transitions required for inflammasome activation. It must be emphasized that this model is entirely hypothesis-generating: all proposed mechanisms remain untested, and experimental validation will be required to establish their biological relevance. Future studies should prioritize the direct experimental interrogation of the IDR-mediated interactions identified here, including co-immunoprecipitation and proximity ligation assays to validate predicted protein-protein contacts, *in vitro* phase separation assays to assess LLPS propensity, mutagenesis of key phosphorylation sites and short linear motifs within IDRs to determine their functional contributions to inflammasome assembly, and hydrogen-deuterium exchange mass spectrometry to characterize IDR conformational dynamics. The IDR-mediated interactions proposed here should be experimentally validated; they may represent candidate targets for strategies aimed at modulating the innate immune responses in inflammation-associated pathologies; however, any translational implications remain speculative at this stage and should not be overstated.

## Data Availability

The original contributions presented in the study are included in the article/**Supplementary Material**. Further inquiries can be directed to the corresponding author.

## Supplementary material

Supplementary Figure 1 Intrinsically Disordered Regions (IDRs) in NLRPs Structures. **(A-K)** Left- Structural diagram of NLRPs indicating IDRs (colored with red) based on disorder score predictions by IUPRED2 and MobiDB. LLPS scores predicted by FuzDrop are also presented. Right- Predicted structure of human NLRPs using Alphafold. The IDRs are highlighted in magenta. **(A)** NLRP1. **(B)** NLRP2. **(C)** NLRP3. **(D)** NLRP5. **(E)** NLRP6. **(F)** NLRP7. **(G)** NLRP8. **(H)** NLRP10. **(I)** NLRP11. **(J)** NLRP13. **(K)** NLRP14.

Supplementary Figure 2 Comparison of NLRP1, NLRP3, and NLRP14 structural predictions generated by AlphaFold 2 and AlphaFold 3. Structural alignments of models predicted by both versions are shown (green: AlphaFold 2; cyan: AlphaFold 3). AlphaFold 2 models were selected for subsequent analyses as they provided a more consistent representation of the intrinsically disordered regions (IDRs), better preserving their extended and flexible characteristics. **(A)** NLRP1. **(B)** NLRP3. **(C)** NLRP14.

Supplementary Figure 3 Predicted interactions of IDRs downstream of the PYD domain of human NLRP1, NLRP3, and NLRP14 with ASC, TXNIP, and TBK1, respectively, as generated by ClusPro. Four representative models were selected from the ten highest-ranked docking models based on the presence of predicted interactions involving the IDRs located downstream of the PYD domain and their corresponding binding partners. **(A)** NLRP1–ASC predicted interaction models. **(B)** NLRP3–TXNIP predicted interaction models. **(C)** NLRP14–TBK1 predicted interaction models.

Supplementary Figure 4 IDRs of NLRP1 exhibit a probability of interaction with BCL2. Interaction models were generated via docking using the HDock Server. Models with a high probability of interaction involving the IDRs were selected for analysis. **(A)** Three Models illustrating three possible interactions between NLRP1 and BCL2. Docking score: -265.50, -259.18, and -253.82, respectively. RMSD with the ligand: 47.30, 59.23 and 60.29 Å. **(B)** Model of the three interactions of NLRP1 and BCL2 rear view.

Supplementary Figure 5 IDR after the PYD domain of NLRP1 exhibits a probability of interaction with BCL-XL. Interaction models were generated via docking using the HDock Server. Models with a high probability of interaction involving the IDRs were selected for analysis. Model illustrating four possible interactions between NLRP1 and BCL-XL. Docking score: -273.42, -264.40, -260.91, and -248.08, respectively. RMSD with the ligand: 60.11, 66.23, 55.57, and 60.29 Å.

Supplementary Figure 6 POP1 structure has intrinsically disordered regions. **(A)** Human POP1 structure predicted by Alphafold, the IDRs are colored in magenta. **(B)** Diagram of the structure of POP1, disorder score predicted by IUPRED2 (IDRs colored by orange square) and MobiDB, and the diagram of the scores of LLPS predicted by FuzDrop.

Supplementary Figure 7 IDR after the PYD domain of NLRP3 exhibits a probability of interaction with ASC and NEK7. Interaction models were generated via docking using the HDock Server. Models with a high probability of interaction involving the IDRs were selected for analysis. **(A)** Three Models illustrating three possible interactions between NLRP3 and ASC. Docking score: -321.47, -248.63, and - 234.79 respectively. RMSD with the ligand: 73.23, 64.70, and 74.64 Å. **(B)** Model demonstrating four possible interactions between IDR1 and IDR2 of NLRP3 and NEK7. Docking score: with IDR1 -255.60, -212.91, and -207.63, with IDR3 -212.28. RMSD with the ligand: with IDR1 79.92, 82.83, and 86.69 Å, with IDR3 48.78 Å.

Supplementary Figure 8 IDRs between NLRP3 and NLRP11 exhibit a probability of interaction. Interaction models were generated via docking using the HDock Server. Models with a high probability of interaction involving the IDRs were selected for analysis. Two models illustrate two possible interactions between NLRP3 and NLRP11 by the IDRs. Green shows NLRP3. The first model shows NLRP11 in yellow, the interaction occurs between several amino acids of the IDR1 of NLRP3 with the IDR2 of NLRP11, docking score: -264.74, RMSD with the ligand 107.87 Å. The second model shows NLRP11 in blue, the interaction occurs between several amino acids of the IDR1 NLRP3 with some amino acids of the PYD domain of NLRP11, and the interaction between several amino acids of the IDR3 NLRP3 with some amino acids of the LRR domain of NLRP11, docking score: -245.84, RMSD with the ligand: M95.19 Å.

Supplementary Figure 9 Structural comparison of phosphorylated and non-phosphorylated NLRP models. Predicted structures generated by AlphaFold 2 in the absence of post-translational modifications (gray) were compared with structures generated by AlphaFold 3 incorporating experimentally reported phosphorylation sites from the PhosphoSitePlus database (green; phosphorylated residues highlighted in red). **(A)** NLRP1. **(B)** NLRP3. **(C)** NLRP14.

Supplementary Figure 10 Effect of phosphorylation on the predicted IDR-mediated interactions in NLRP1 and NLRP3. Computational docking models illustrating predicted interactions between phosphorylated NLRP structures and their respective binding partners, as generated by HDOCK. **(A)** Predicted interaction between NLRP1 and ASC. NLRP1 is shown in brown; each color represents a distinct docking model. The light green ASC model predicts interaction with IDR1, while the remaining models predict interaction with IDR2. **(B)** Predicted interaction between NLRP3 and TXNIP. NLRP3 is shown in brown; each color represents a distinct docking model. The light green TXNIP model predicts interaction with IDR2, while the remaining models predict interaction with IDR3.

Supplementary Figure 11 DDX3X family members in humans have IDRs like DDX3X. Prediction of the IDRs (colored in orange) and other characteristics in the 18 members of the DDX3X family in humans. Predictions were obtained from the D2P2 database.

Supplementary Table 1 Phosphorylation sites in NLRP1, NLRP3 and NLRP14.

Supplementary Table 2 Mutations in intrinsically disordered regions of NLRP1, NLRP3, and NLRP14 relative to structured (crystallized) domains.
