# Supplementary material for: Intrinsically disordered regions in the NLRP family of receptors act as regulatory hubs for inflammasome condensation and activation
Source: Front Immunol. 2026 Sep 1;17:1793320. doi: 10.3389/fimmu.2026.1793320 (PMC13574816; doi:10.3389/fimmu.2026.1793320)
Supplement: Supplementary file 1 [file SupplementaryFile1.docx]

**Reference**

1. Casado P, Alcolea MP, Iorio F, Rodriguez-Prados JC, Vanhaesebroeck B, Saez- Q23 Rodriguez J, et al. Phosphoproteomics data classify hematological cancer cell lines according to tumor type and sensitivity to kinase inhibitors. Genome Biol. (2013) 14: R37. doi: 10.1186/gb-2013-14-4-r37

2. Jenster LM, Lange KE, Normann S, vom Hemdt A, Wuerth JD, Schiffelers LDJ, et al. P38 kinases mediate NLRP1 inflammasome activation after ribotoxic stress response and virus infection. J Exp Med. (2023) 220. doi: 10.1084/jem.20220837

3. Mertins P, Yang F, Liu T, Mani DR, Petyuk VA, Gillette MA, et al. Ischemia in tumors induces early and sustained phosphorylation changes in stress kinase pathways but does not affect global protein levels. Mol Cell Proteomics. (2014) 13:1690–704. doi: 10.1074/mcp.m113.036392

4. Hornbeck PV, Zhang B, Murray B, Kornhauser JM, Latham V, Skrzypek E. PhosphoSitePlus, 2014: mutations, PTMs and recalibrations. Nucleic Acids Res. (2015) 43:D512–520. doi: 10.1093/nar/gku1267

5. Stutz A, Kolbe CC, Stahl R, Horvath GL, Franklin BS, van Ray O, et al. NLRP3 inflammasome assembly is regulated by phosphorylation of the pyrin domain. J Exp Med. (2017) 214:1725–36. doi: 10.1084/jem.20160933

6. Zhao W, Shi CS, Harrison K, Hwang IY, Nabar NR, Wang M, et al. AKT regulates NLRP3 inflammasome activation by phosphorylating NLRP3 serine 5. J Immunol.(2020) 205:2255–64. doi: 10.4049/jimmunol.2000649

7. Song N, Li T. Regulation of NLRP3 inflammasome by phosphorylation. Front Immunol. (2018) 9:2305. doi: 10.3389/fimmu.2018.02305

8. Dufies O, Doye A, Courjon J, Torre C, Michel G, Loubatier C, et al. Escherichia coli Rho GTPase-activating toxin CNF1 mediates NLRP3 inflammasome activation via p21-activated kinases-1/2 during bacteraemia in mice. Nat Microbiol. (2021) 6:401–12. doi: 10.1038/s41564-020-00832-5

9. Niu T, De Rosny C, Chautard S, Rey A, Patoli D, Groslambert M, et al. NLRP3 phosphorylation in its LRR domain critically regulates inflammasome assembly. Nat Commun. (2021) 12:5862. doi: 10.1038/s41467-021-26142-w

10. Wu R, Hogberg J, Adner M, Ramos-Ramirez P, Stenius U, Zheng H. Crystalline silica particles cause rapid NLRP3-dependent mitochondrial depolarization and DNA damage in airway epithelial cells. Part Fibre Toxicol. (2020) 17:39. doi: 10.1186/s12989- 020-00370-2

11. Mortimer L, Moreau F, MacDonald JA, Chadee K. NLRP3 inflammasome inhibition is disrupted in a group of auto-inflammatory disease CAPS mutations. Nat Immunol. (2016) 17:1176–86. doi: 10.1038/ni.3538

12. Mezzasoma L, Talesa VN, Romani R, Bellezza I. ANP and BNP exert anti-inflammatory action via NPR-1/cGMP axis by interfering with canonical, non-canonical, and alternative routes of inflammasome activation in human THP1 cells. Int J Mol Sci. (2020) 22. doi: 10.3390/ijms22010024

13. Guo C, Xie S, Chi Z, Zhang J, Liu Y, Zhang L, et al. Bile acids control inflammation and metabolic disorder through inhibition of NLRP3 inflammasome. Immunity. (2016) 45:944. doi: 10.1016/j.immuni.2016.09.008

14. Spalinger MR, Schwarzfischer M, Scharl M. The role of protein tyrosine phospha- tases in inflammasome activation. Int J Mol Sci. (2020) 21. doi: 10.3390/ijms21155481

15. Nucleic Acids Research, 29(1), 308–311. https://doi.org/10.1093/nar/29.1.308

16. Sherry, S. T., Ward, M., & Sirotkin, K. (1999). dbSNP—Database for single nucleotide polymorphisms and other classes of minor genetic variation. Genome Research, 9(8), 677–679. doi.org
