# Supplementary material for: Intrinsically disordered regions in the NLRP family of receptors act as regulatory hubs for inflammasome condensation and activation
Source: Front Immunol. 2026 Sep 1;17:1793320. doi: 10.3389/fimmu.2026.1793320 (PMC13574816; doi:10.3389/fimmu.2026.1793320)
Supplement: Supplementary file 2 [file Table1.docx]

**Table S1. Phosphorylation sites in NLRP1, NLRP3 and NLRP14**

|  | PTM | IDR/Domain | Disease tissue/cell type | IDR structure modification | Reference |
| --- | --- | --- | --- | --- | --- |
| NLRP1 | T41-p | PYD | Acute monoblastic leukemia (M5a).  Acute monocytic leukemia (M5b).  Acute myeloblastic leukemia, without maturation (M1).  B cell lymphoma.  Non-Hodgkin's lymphoma.  Multiple myeloma. |  | [1] |
|  | S107-p | IDR1 | HEK293T (epithelial). |  | [2] |
|  | T240-p | IDR2 | HEK293T (epithelial). |  | [2] |
|  | S291-p | NACHT | Ischemia. |  | [3] |
|  | S823-p | FIND | Lung cancer: non-small cell lung cancer. |  | [4] |
|  | T993-p | IDR3 | HEK293T (epithelial). |  | [2] |
|  | T1000p | IDR3 | Brain cancer: glioblastoma, glioma. |  | [4] |
|  | S1017-p | IDR3 | HEK293T (epithelial). |  | [2] |
|  | S1022-p | CARD | HEK293T (epithelial). |  | [2] |
|  | S1371-p | CARD | HEK293T (epithelial). |  | [2] |
| NLRP3 | S5-p | PYD | Activity inhibited.  HEK293T (epithelial).  Macrophage.  BMDM. |  | [5, 6] |
|  | Y13-p | PYD | HEK293T (epithelial). |  | [7] |
|  | S161-p | FISNA | HEK293T (epithelial).  Macrophage. |  | [5] |
|  | S163-p | FISNA | HEK293T (epithelial) |  | [7, 8] |
|  | S198-p | IDR2 | Lymphoma.  HEK293T (epithelial).  U-937 (myeloid). |  | [8-10] |
|  | T233-p | NACHT | Esophageal cancer. |  | [7] |
|  | S295-p | NACHT | Leukemia  Acute myelogenous leukemia. |  | [11-13] |
|  | S334-p | NACHT | HEK293T (epithelial). |  | [7] |
|  | S387-p | NACHT | Liver cancer. |  | [4] |
|  | S436-p | HD1 | Jurkat (T lymphocyte). |  | [4] |
|  | T659-p | IDR3 | HEK293T (epithelial). Macrophage. | Alpha helix | [8] |
|  | S728-p | HD2 | HEK293T (epithelial).  Macrophage. |  | [4] |
|  | S735-p | HD2 | Lymphoma.  HEK293T (epithelial).  U-937 (myeloid). |  | [9] |
|  | S806-p | LRR | Lymphoma.  HEK293T (epithelial).  U-937 (myeloid).  HeLa (cervical). |  | [9] |
|  | Y861-p | LRR | Leukemia, acute myelogenous leukemia, multiple myeloma |  | [14] |
|  | S975-p | LRR | HEK293T (epithelial) |  | [4] |
|  | S1035-p | LRR | Lymphoma.  HEK293T (epithelial),  U-937 (myeloid) |  | [9] |
| NLRP14 | T101-p | IDR2 | Brain cancer: Glioblastoma, Glioma. | Alpha helix | [4] |
|  | S302-p    T310-p | NATCH | Lung cancer, surrounding tissue. |  | [4] |
|  |  | NATCH | Jurkat (T lymphocyte). |  | [4] |
|  | T311-p | NATCH | Jurkat (T lymphocyte). |  | [4] |
|  | S312-p | NATCH | Jurkat (T lymphocyte). |  | [4] |
|  | Y443-p | NOD2 | Jurkat (T lymphocyte). |  | [4] |
|  | T1022-p | LRR | NCI-H3255 (pulmonary). |  | [4] |
|  | S1040-p | LRR | NCI-H3255 (pulmonary). |  | [4] |
