# Supplementary material for: Intrinsically disordered regions in the NLRP family of receptors act as regulatory hubs for inflammasome condensation and activation
Source: Front Immunol. 2026 Sep 1;17:1793320. doi: 10.3389/fimmu.2026.1793320 (PMC13574816; doi:10.3389/fimmu.2026.1793320)
Supplement: Supplementary file 3 [file Table2.docx]

**Table S2. Mutations in intrinsically disordered regions of NLRP1, NLRP3, and NLRP14 relative to structured (crystallized) domains [15,16].**

|  | Mutation | IDR | ΔΔG* | Location in structure | Pathology |
| --- | --- | --- | --- | --- | --- |
| NLRP1 | L155Q | IDR2 | 0.43 | Surface | Vitiligo-associated multiple autoimmune disease 1 (VAMAS1) |
| NLRP3 | R167Q | IDR2 | 0.33 | Surface | Syndrome de Von |
|  | I174T | IDR2 | 0.61 | Surface | Chronic infantile neurological cutaneous and articular syndrome (CINCA) |
| NLRP14 | R84L | IDR1 | 0.03 | Inter | No pathology reporter. There is greater thermal stability of the pyrin domain. |
|  | D86V  K92R | IDR1 | 0.03  -0.19 | Surface | Spermatogenic failure |
|  |  | IDR1 |  | Surface | Benign |
|  | S98L |  | 0.09 | Surface | Not reported |

* We consider variants without structural changes whose ΔΔG values are in the range between -0.5 and 0.5 kcal/mol.
